# A Generic Approach for Miniaturized Unbiased High-Throughput Screens of Bispecific Antibodies and Biparatopic Antibody–Drug Conjugates

**DOI:** 10.3390/ijms25042097

**Published:** 2024-02-08

**Authors:** Nadine Barron, Stephan Dickgiesser, Markus Fleischer, Angelika-Nicole Bachmann, Daniel Klewinghaus, Jens Hannewald, Elke Ciesielski, Ilja Kusters, Til Hammann, Volker Krause, Sebastian Winfried Fuchs, Vanessa Siegmund, Alec W. Gross, Dirk Mueller-Pompalla, Simon Krah, Stefan Zielonka, Achim Doerner

**Affiliations:** 1 Protein and Cell Sciences, EMD Serono, 45A Middlesex Turnpike, Billerica, MA 01821, USA; 2 NBE Technologies, Merck Healthcare KGaA, Frankfurter Str. 250, 64293 Darmstadt, Germany; 3 Protein Engineering and Antibody Technologies, EMD Serono, 45A Middlesex Turnpike, Billerica, MA 01821, USA; 4 Discovery Pharmacology, Merck Healthcare KGaA, Frankfurter Str. 250, 64293 Darmstadt, Germany

**Keywords:** DuoBody, bispecific antibody, unbiased screening, automation, miniaturization

## Abstract

The toolbox of modern antibody engineering allows the design of versatile novel functionalities exceeding nature’s repertoire. Many bispecific antibodies comprise heterodimeric Fc portions recently validated through the approval of several bispecific biotherapeutics. While heterodimerization methodologies have been established for low-throughput large-scale production, few approaches exist to overcome the bottleneck of large combinatorial screening efforts that are essential for the identification of the best possible bispecific antibody. This report presents a novel, robust and miniaturized heterodimerization process based on controlled Fab-arm exchange (cFAE), which is applicable to a variety of heterodimeric formats and compatible with automated high-throughput screens. Proof of applicability was shown for two therapeutic molecule classes and two relevant functional screening read-outs. First, the miniaturized production of biparatopic anti-c-MET antibody–drug conjugates served as a proof of concept for their applicability in cytotoxic screenings on tumor cells with different target expression levels. Second, the automated workflow enabled a large unbiased combinatorial screening of biparatopic antibodies and the identification of hits mediating potent c-MET degradation. The presented workflow utilizes standard equipment and may serve as a facile, efficient and robust method for the discovery of innovative therapeutic agents in many laboratories worldwide.

## 1. Introduction

The recent approval of several bispecific antibodies with versatile modes of action has indicated the profound benefit for patients that the educated engineering of proteins and antibodies can have [1,2,3,4,5]. All bispecific and biparatopic antibodies (bsAbs) or antibody–drug conjugates (ADCs) comprise binding modules in combinations not found in nature [6]. In addition, most bispecific antibodies are designed with a heterodimeric Fc portion, although higher valency symmetric formats exist as well [7]. Several methodologies have been reported for the generation of developable heterodimeric Fc antibodies, and more are becoming clinically validated [8,9,10,11]. While optimization of known binder combinations and production of thus optimized lead candidates can be challenging, the choice of suitable formats and improved development processes have increased the success rates during these steps [7]. However, as the combination of the best parental binders is not necessarily the best combination for each functionality, the identification of paratope pairs eliciting a desired mode of action (MoA) in the best possible way remains a major challenge [12]. 

**Figure 1:**
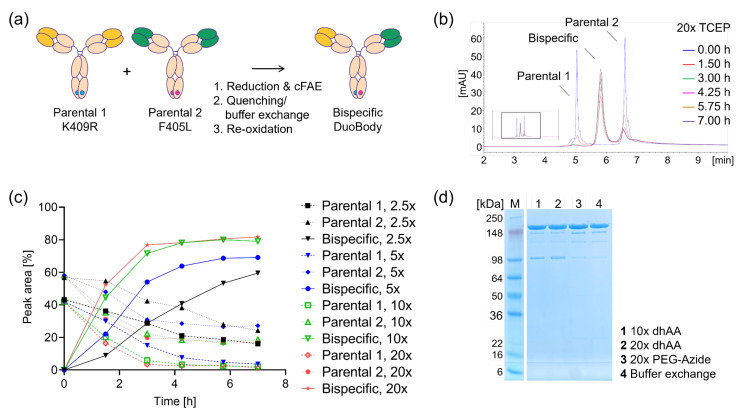
Optimization of miniaturized cFAE via TCEP and re-oxidation, applying dhAA or PEG-azide. (**a**) Scheme of the DuoBody reaction for heterodimerization. (**b**) Exemplary raw data set from SCX-HPLC monitoring cFAE over time, upon the addition of a 20-fold molar excess of TCEP at 1 mg/mL. Please refer to supplementary Figure S1 indicating a negligible peak caused by PEG-azide. (**c**) cFAE kinetics for four TCEP concentrations applying a representative parental DuoBody pair to optimize TCEP concentration and incubation time. (**d**) Re-oxidation of interchain disulfide bonds using 10- or 20-fold dhAA or 20-fold PEG-azide (molar access over TCEP) compared to buffer exchange. PEG-azide yielded the formation of >90% species of the size expected for the re-oxidized antibody similar to buffer exchange, confirmed by CE-SDS, as shown in Figure S2. Numerical data for sections (**b**,**c**) are listed in Table S1.

Nowadays, antibody selection campaigns such as immunization combined with B-cell cloning or yeast surface display typically yield diverse parental antibody panels in the range of up to several hundred initial hit candidates [13]. The production and testing of every combination require a stable high-throughput (HTP) compatible bsAb production process for several thousand different molecules. Although reported to be feasible by Kitazawa and co-workers [14] for over 40,000 bispecific antibody combinations, the production of parental binders and the establishment of a protein-based heterodimerization methodology would save production capacities and time. The production of 200 + 200 = 400 parentals would enable the screening of 200 × 200 = 40,000 combinations. In comparison to the plethora of multispecific formats and therapeutic candidates in preclinical and clinical development, few approaches exist to overcome the bottleneck of combinatorial screening. In addition to HTP cloning and small-scale expression [15], technologies such as paired-light chain single-cell production [16], the exemplary B-Body format [17], the SpyTag/SpyCatcher system [18,19], split inteins [20,21], Fab-based systems [22] or the ForCE process [23] were adapted to enable broad bispecific antibody screens. Also, controlled Fab-arm exchange (cFAE), the so-called DuoBody reaction, has been applied at different concentrations and setups [24,25,26] for the production of therapeutics as well as initial hit identification from few combinations [4,27]. Prerequisite for cFAE are two separately produced parental antibodies comprising an F405L or a K409R mutation within the CH3 domain. Activation of the reaction mix via mild reduction and re-oxidation yields a high percentage of heterodimeric bispecific antibodies (Figure 1a). As a specific subtype of bispecifics, antibodies binding with two different paratopes to the same target (biparatopics) have been reported to mediate enhanced functionalities such as high-order crosslinking leading to the internalization and degradation of target proteins and antibodies [28,29,30,31]. This mode of action has lately been exploited for both enhanced degradation of oncogenic c-MET for anti-tumor treatment [29] as well as enhanced payload delivery of highly potent ADC approaches [28,32]. Similar to the challenges in hit discovery discussed above, also the identification of paratopes for inter- versus intra-target binding eliciting optimal target degradation remains a challenge (Evers A., mAbs 2023, in review) [33].

We here report, to our knowledge for the first time, a miniaturization and full automation procedure to make cFAE amendable for the HTP combinatorial screening of bispecific antibodies and ADCs. As a proof-of-concept study, we applied the identified procedures to the small-scale production of biparatopic anti-c-MET ADCs enabling cytotoxicity screens with tumor cells of varying c-MET expression levels. In a second study, the automated workflow enabled a large unbiased combinatorial screening of biparatopic antibodies and the identification of hits mediating potent c-MET degradation. These two different applications indicate their general applicability for the unbiased high-throughput screening (HTS) of optimal bispecific or biparatopic antibodies.

## 2. Results

### 2.1. HTS-Compatible cFAE Setup

The optimization of cFAE protocols amendable for later high-throughput screens was conducted with four combinations of exemplary internal parental DuoBody antibodies covering several formats and with varying efficiency of cFAE in the published mid-scale process [25]. The antibody pair resulting in the lowest heterodimer content was chosen for subsequent small-scale optimization (Figure 1) to ensure applicability to a high share of future combinations. In a first step, cFAE kinetics were optimized for TCEP as a reducing agent, based on previous reports of its applicability for cFAE [25]. Reactions with 1 mg/mL assessed by SCX HPLC were revealed to be optimal with incubation with a 20-fold molar excess of TCEP for 4 h for complete cFAE (Figure 1b,c). Please note the uneven molar ratio of the parental antibodies resulting in residual mAb2, indicating the need for equimolar mixing rather than matching protein concentrations calculated in mg/mL. 

In a second step, the reformation of interchain disulfide bonds covalently linking the DuoBody chains was evaluated. The addition of dehydroascorbic acid (dhAA) or PEG-azide for the quenching of residual TCEP and disulfide oxidation were compared against the more laborious exchange of buffer. While dhAA enabled the formation of 85–87% stable heterodimer, the application of PEG-azide was as efficacious as buffer exchange, with a stable heterodimer content greater than 90%, as shown by denaturing SDS-PAGE and CE-SDS (Figure 1d and Figure S2). Although this protocol was confirmed not to negatively influence functionality, minor additional SDS-PAGE bands (Figure 1d) indicated the presence of potentially disulfide scrambled fractions that were minimized by applying the lowest effective TCEP concentration possible. In summary, a simple workflow at ambient temperature consisting in mixing parental DuoBodies at equimolar 1 mg/mL concentration, the addition of a 20-fold TCEP excess followed by a 4 h incubation, and the subsequent addition of a 40-fold concentration of PEG-azide for re-constitution was found optimal and suitable for miniaturization and automation.

### 2.2. cFAE Optimization for Low Volumes or Concentrations

**Figure 2:**
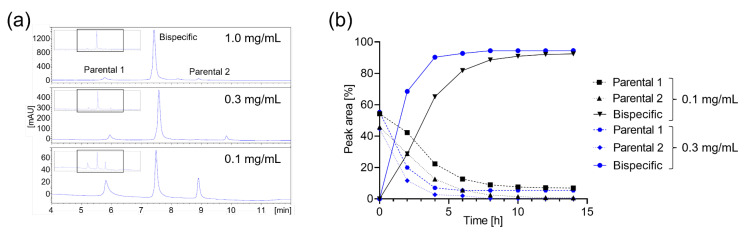
Concentration and time dependency of the miniaturized reaction. (**a**) HIC HPLC analyses of the cFAE reaction with a 20-fold molar excess of TCEP for 4 h at 1 mg/mL, 0.3 mg/mL and 0.1 mg/mL, revealing a clear concentration dependency. (**b**) cFAE time course at 0.3 mg/mL and 0.1 mg/mL indicating slower reaction kinetics but completion at >12 h incubation times. The working condition consisting of 0.7–1 mg/mL in 50–100 µL reaction volume and 4 h of cFAE reaction was found optimal. Numerical data for section (**b**) are listed in Table S2.

The concentration of 1 mg/mL of most antibody formats equals micromolar concentrations often not needed for bispecific antibody primary screens. High concentrations in high volumes and a large number of combinations would require high quantities of parental antibodies that are often not available during the screening stage. As a reduction in reaction volume is limited, we next aimed at the evaluation of cFAE efficiency at lower concentrations. cFAE reactions at 0.3 or 0.1 mg/mL showed substantially slower reaction kinetics (Figure 2a) that could be counteracted by longer incubation times (Figure 2b). As incubation at higher temperatures or for longer times was deemed inappropriate due to potential evaporation, using 0.7–1 mg/mL in 50–100 µL was determined to provide optimal working concentration, volume and resulting material amount for subsequent screens.

### 2.3. Automated Combinatorial Pipetting and cFAE

To reduce the error rates and enable a scalable process, matrix pipetting was realized on both Tecan and Hamilton liquid handlers. Assuming an average molecular weight (MW) of 145.3 kDa or 78.5 kDa for the parental IgG1 or VHH-Fc fusions, respectively, the resulting heterodimeric bsAbs would have an average MW of 111.9 kDa. The VHH-Fc volumes added were therefore adapted by a factor of 0.54 to yield a 1:1 stoichiometric ratio with respect to the IgG parentals. A 9 × 9 combinatorial pilot run on a Hamilton device with exemplary parentals appropriate for full analytics confirmed a high heterodimer content in randomly picked samples. To evaluate the applicability for full high-throughput screens, 24 × 26 = 624 combinations of anti-c-MET biparatopic antibodies were generated on a Tecan liquid handler (Fluent 780) within 1 h. The heterodimer content was confirmed to be high in randomly picked samples (Figure S3). In mixed samples with a lower resulting heterodimer content, clearly one parental antibody was limited, but cFAE was complete, indicating both the robustness of the established methods and the need for strictly equimolar mixing. The application of the generated biparatopic antibodies in a high-throughput functional screen for target degradation is presented in the next paragraph. Limited by the parental sample numbers in this application, the theoretical throughput of one run in the established assay-ready setup would be 1260 combinations including the controls (7 rows × 9 columns × 20 96-well plates) generated in 2 h, fully supporting most bispecific or biparatopic screening needs. Additional capacity could be realized using full plates (8 × 12 × 20 plates = 1920 combinations), by increasing the possible number of plates and the option to repeat runs.

### 2.4. Applicability for Biparatopic ADC Screening

**Figure 3:**
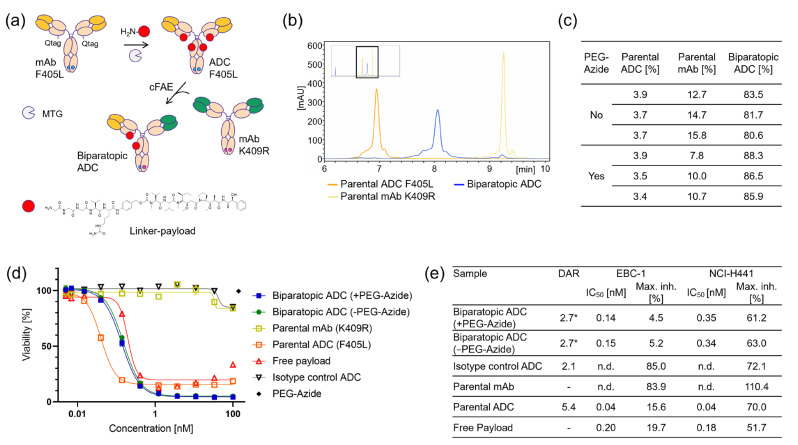
Applicability for biparatopic ADC screening. (**a**) Scheme of the preparative steps including MTG-mediated linker–payload conjugation and heterodimerization to yield “half-ADC” candidates amendable for functional interrogation. (**b**) Representative CIEX-HPLC chromatograms indicating the successful generation of an REGN5093-based antibody–ADC heterodimer/biparatopic ADC (bpADC) applying the conditions optimized for bispecific antibody generation. (**c**) Triplicate analyses of biparatopic ADC formation at 1 mg/mL, 40 eq. TCEP and 6 h incubation time (please refer also to Figure S3). (**d**) Applicability of the generated half ADCs for functional screening such as for cytotoxic potency. Please note that the higher potency of the parental ADC is due to a higher DAR. (**e**) Potency determination of the samples shown in (**d**), demonstrating the option to screen for functionality with a DAR2 biparatopic ADC. * Indicates the calculated DAR values.

In addition to unconjugated bispecific antibody approaches, bispecific ADCs (i.e., biparatopics) have recently been reported as novel potent therapeutic modalities. However, the identification of optimal paratope pairs for enhanced payload delivery and ADC efficacy remains challenging. To evaluate the option to screen for effective biparatopic ADCs, the miniaturized cFAE process was further adapted and evaluated in the formation of DuoBody ADCs. In order to reduce the number of required conjugation reactions in HTP approaches, we anticipated the formation of “half ADCs” by combining parental ADCs with parental unconjugated antibodies (Figure 3a). For this, a suitable anti-c-MET parental REGN5093 reference antibody [32] was equipped with a cytotoxic MMAE payload using enzymatic microbial transglutaminase (MTG) bioconjugation [34]. Contrary to the anticipated DAR of 4, around 5.4 drugs were conjugated per antibody due to a free glutamine residue in the kappa light chain. The optimization of the heterodimerization reaction between pre-conjugated ADC and antibody parentals revealed efficient cFAE at 1 mg/mL of mAb/ADC and 40 eq. TCEP after 6 h, with slightly higher heterodimer contents in reactions with of PEG-azide (Figure 3b,c and Figure S4). The resulting half-conjugated biparatopic ADCs inherited a calculated halved DAR that was shown to be sufficient to evoke a c-MET expression-dependent cytotoxic effect on target cells (Figure 3d,e). As this setup comprises a widely established, readily applicable assay and read-out, this approach appears to be suited to screening for potent next-generation ADCs.

### 2.5. High-Throughput Functional Bispecific Screening

**Figure 4:**
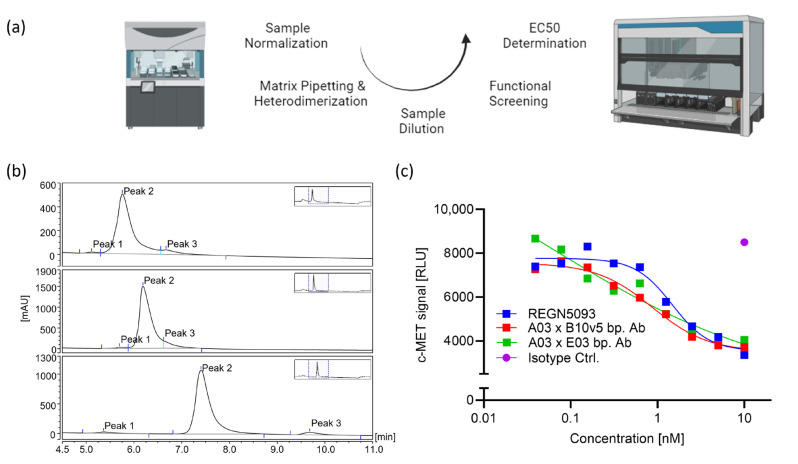
Heterodimerization and c-MET degradation pilot study. (**a**) Scheme for automated matrix pipetting and heterodimerization followed by high-throughput functional interrogation and hit confirmation. (**b**) Representative HIC-HPLC chromatograms for 3 out of 624 c-MET biparatopic combinations. Marked peaks refer to bispecifics (Peak 2) and their respective parental antibodies (Peak 1, Peak 3) and indicate a high heterodimer content. Please, see also Figure S3 for a broader data panel. (**c**) High-throughput screening for c-MET degradation yielded several potent combinations. Representative data indicate the nanomolar c-MET degradation potency (IC50 values: 0.91 nM, A03xB10v5; 0.80 nM, A03xE03; 1.47 nM, REGN5093) of two novel biparatopic antibodies comprising VHH A03 in combination with Fabs B10v5 or E03, similar to that of the re-produced REGN5093 reference, while the anti-DIG IgG isotype control did not degrade the antigen.

To evaluate a mechanistically similar but biologically different mode of action relevant for another group of novel therapeutic antibodies, a biparatopic antibody screening for efficient degradation of the oncogenic driver antigen c-MET was established and applied for the identification of optimal paratope combinations (Figure 4a). Parental antibodies were chosen from IgG1 and VHH-Fc fusion panels and pre-selected for purities > 95%, qualified specific cellular binding, and absence of in silico developability flags such as post-translational modifications (PTMs). No further filtering such as epitope binning was applied to enable a fully unbiased screening for the best combination. The generation of biparatopics was extended by inclusion of an isotype control arm for each combination and in-process generation of the reference antibody REGN5093, summing up to 624 combinations. Assay qualification including a signal window >3 for isotype to inhibitor controls, CV < 0.2 at maximal, median and minimal efficacy values of the REGN5093 reference, as well as Z′ > 0.45 confirmed the applicability of HTP screening for c-MET degradation. Successful automated biparatopic generation with high heterodimer contents (as indicated by the major “Peak 2” in Figure 4b and Figure S3) and a robust automated screening assay setup enabled the identification of several biparatopic hits with high c-MET degradation potency (Figure 4c), demonstrating the applicability of the developed miniaturized and automated cFAE process for functional bispecific or biparatopic screens. 

## 3. Discussion

Following the validation of monoclonal antibodies as efficacious and safe therapeutic agents, research in recent years has underscored the substantial prospects of bispecific antibodies (bsAb) to further extend therapeutic intervention options beyond those allowed by the toolbox found in nature. BsAbs possess the remarkable ability to simultaneously target two distinct antigens, offering novel therapeutic modes of action, including enhanced selectivity [35] or efficacy [4], reduced resistance and expanded treatment possibilities in oncology, autoimmune disorders and infectious diseases. While hit discovery processes have been optimized to effectively identify monoclonal antibodies with the optimal desired functionality, the combinatorial nature of bispecific antibodies poses a challenge when aiming at the discovery of the best possible combination from two broad parental monospecific antibody panels [36]. Although in some cases the desired functionality can be obtained via the engineering of a given but potentially suboptimal combination [35], optimal paratope combinations often need to be identified within a large screening space. Within those, hit rates often lie in a 0.1–1% range [14,22,23,37], illustrating the apparent need for unbiased combinatorial high-throughput screens to yield optimal paratope pairs mediating the desired therapeutic functions. This can be accomplished by highly paralleled manual screening [14], automated high-throughput cloning and production [15,16,37], library approaches in combination with microfluidic-assisted functional sorting [38,39,40] or bispecific screening after heterodimerization on the protein level [21,22,23]. 

Focusing on heterodimeric Fc bispecifics as a major therapeutically applied format, we utilized controlled Fab-arm exchange (cFAE), known in colloquial language as the DuoBody reaction, due to its several advantages over the options referenced above, i.e.,: (1) the ease of parental antibody production applying standard IgG production procedures; (2) the opportunity to evaluate affinity, epitope bin, cellular binding, internalization, physico-chemical properties such as thermal stability or hydrophobicity, polyspecificity or self-interaction in IgG format with the same material in advance or in parallel to an HTP screening; (3) the high degree of heterodimerization; (4) the applicability for bsAb, but also biparatopic, ADC screening as reported herein. cFAE has been identified in human IgG4 antibodies [26,41,42,43], extended to therapeutic bsAb production applying MEA as a reducing agent [25] and extensively studied, including the evaluation of MEA versus DTT or cFAE kinetics [44] and already applied in focused combinatorial screens [27], leading, among others, to the recently approved bispecific antibody amivantamab [4]. Although this is illustrative of an elegant, educated screening cascade based on deep biological knowledge, many novel modes of action necessitate complex and high-throughput screening setups targeting unprecedented biology without an a priori definition of optimal epitope combinations or their respective paratopes. We therefore developed a robust miniaturized heterodimerization process applicable for high-throughput functional screening based on cFAE with suitable optimized reaction conditions.

The novel setup applying TCEP and PEG-azide is compatible with automated high-throughput screens by reagent addition rather than the laborious buffer exchange and yields high proportions of the desired heterodimers (Figure 3, Figure 4 and Figure S3). Based on previous reports of several reducing agents being suitable for cFAE initiation (Figure S2 in Labrijn et al., 2013 [25]), TCEP was chosen due to its high activity at low concentrations, enabling its quenching by oxidants at concentrations compatible with subsequent biological screens. At a 1 mg/mL antibody concentration, a 20-fold molar excess of TCEP over the parental antibodies was found optimal. To allow the re-formation of disulfide bonds covalently connecting the antibody chains, PEG-azide and dhAA, both reported to quench TCEP, and the latter typically used for active disulfide re-oxidation [45,46,47], were evaluated as alternatives to the labor-intensive buffer exchange. PEG-azide was chosen over dhAA due to the higher final content of species of the expected size in SDS-PAGE and CE-SDS, indicating successful disulfide reformation (Figure 1d and Figure S2). Optimization was achieved with the application of a 40-fold molar excess and incubation at ambient temperature for several days to ensure full re-oxidation. Minimal disulfide-bridge scrambling (Figure 1d) was observed in all samples, was the lowest in the PEG-azide setup and was assumed not to negatively influence antibody binding or function. In the assays evaluated so far, TCEP and PEG-azide were found not to interfere with the assay read-out (Figure 3d and Figure 4), but it is advisable to confirm this observation during the development of other screening assays.

When trying to reduce the concentration of the parental antibodies to the minimize material demand for larger combinatorial screens, a concentration dependency was observed, analogous to previous reports [44]. As elongating the cFAE incubation period would have compromised the operational timeline, a final optimal concentration range from 0.5 to 1 mg/mL was set. However, in the case of a limited availability of the parental antibodies or very high combinatorial numbers, lower concentrations and longer incubation times would still represent a feasible setup.

For assay setups more sensitive to the remaining parental monomer amounts, close monitoring of the heterodimer content is warranted. Equimolar ratios were desired and here realized by adapting the volumes, assuming average molecular weights. Please note that adjusting to MW differences is feasible for both smaller or larger combination partners, but a slight MW deviation from the average by individual combination partners can lead to an imbalanced parental ratio and a subsequently reduced heterodimer content (see Figure 1c). Normalization to the same molarity rather than to a “mg/mL” protein concentration could help to reduce the parental content and represents a generalized option for the heterodimerization of diverse samples of different sizes and sources. In the study presented herein, deviation was less than 5% and regarded as acceptable. As shown in Figure 1, Figure 2, Figure 3 and Figure 4, the workflow is applicable to IgG-like bispecifics but also to VHH/Fab combinations, and application to further heterodimeric formats such as IgG/IgG–VHH [48] is conceivable. The resulting bsAb hit combinations could be genetically re-constructed in the heterodimeric Fc format of choice such as the knob-into-hole format for orthogonal hit validation. Although cFAE has been reported to be efficient at different scales [25], this study represents, to our best knowledge, the first report of an optimized miniaturized cFAE workflow amendable for high-throughput screening.

Similar to bsAbs, biparatopic antibodies and antibody–drug conjugates (bpADCs) exhibit substantial potential for therapeutic interventions [28,49]. Suitable paratope combinations can increase target cross-linking, boost internalization and enhance degradation [29,50], as well as improve payload delivery when applied as bpADCs. Nonetheless, the identification of appropriate binder pairs facilitating these desired functions presents a nontrivial challenge that can be overcome by high-throughput functional screening methodologies. As options for robust high-throughput conjugation yielding comparable drug-to-antibody ratios (DARs) are limited, we evaluated the heterodimerization of pre-conjugated ADC parentals with unconjugated combination partners. In a proof-of-concept study, the applicability for biparatopic ADC formation was shown using a parental c-MET reference antibody [29] conjugated with MMAE using MTG prior to miniaturized heterodimerization with the appropriate antibody counterpart, resulting in a DAR of 2.7 for c-MET bpADCs (Figure 4a–c). The confirmation of target-dependent cytotoxic functionality in several c-MET-expressing cell lines demonstrated the generic potential for screening such “half-conjugated” bpADCs (Figure 3). Frequently, combinatorial screenings encompass one relatively small set of parental antibodies which can be utilized for pre-conjugation, leading to a substantial reduction in laboratory efforts. As an illustrative example, a combinatorial repertoire of 10 × 50 = 500 bpADCs could be subjected to screening by employing merely 10 pre-conjugated parental antibodies. As cytotoxic assays are widely established in ADC laboratories, this methodology should be well suited for effective screening applications identifying potent next-generation ADCs.

The optimized protocol for DuoBody generation was finally applied in a biologically related but methodologically different setup: a large combinatorial set of anti-c-MET biparatopic antibodies was generated and screened for efficient and potent target degradation. The development of the Lumit-based c-MET degradation assay was facilitated by the usage and IC50 determination of the internally small-scale re-produced reference antibody REGN5093 [29], pointing towards screening at one-digit nanomolar concentrations. Target degradation higher than 50% at the REGN5093 IC50 concentration indicated potentially enhanced functional degrading potency and allowed for primary hit definition. The success of high-throughput heterodimerization was confirmed via HIC HPLC of randomly picked samples showing ~80–96% heterodimer content (Figure 4b and Figure S3), which was deemed sufficient for application in a target degradation assay setup. Several c-MET-degrading biparatopic antibodies were identified with similar potency as that of the re-produced REGN5093 reference (Figure 4c). Although tested for 24 × 26 = 624 combinations due to the relatively limited number of available parental antibodies, the automated setup could support larger high-throughput screens applying diverse functional read-outs such as conditional agonism, effector cell recruitment or selectivity by avidity binding.

In the study presented here, the parental antibodies were qualified to be devoid of developability flags such as post-translational modifications and to have purity >95% and were confirmed to be able of specific cellular binding, but no further filtering such as epitope binning was applied to enable a fully unbiased screening. Pre-filtering for good developability and optimal analytical capacity by, e.g., distinct HIC-, CIEX- or RP-HPLC profiles would enhance the probability for a good manufacturability profile and a facile differentiation between parentals and bispecific product. On the other hand, it could strongly reduce diversity and miss the global optimum for the best possible combination. A broad unbiased screen covering a broader screening space would enable an informed choice of few optimal lead bispecifics and their optional sequence optimization for suitable manufacturability along the development path.

In contrast to the proprietary methodologies described in the introduction, the workflow presented here offers several advantages. In fact, widely established small-scale production and purification processes can be applied to cover the need of low milligram amounts of parental antibodies. In addition, only commercially available reagents and standard equipment are utilized with this procedure, which, therefore, represents a generic approach that may serve as a facile, efficient, and robust method for the discovery of novel optimized heterodimeric bispecific or biparatopic biotherapeutics.

## 4. Material and Methods

### 4.1. Manual cFAE

The optimization of cFAE protocols amendable for later high-throughput screens was conducted with four exemplary internal DuoBody combinations covering several formats and a range of good-to-poor performing pairs regarding cFAE and re-oxidation when applying the initially published cFAE process [25]. The combination resulting in the lowest heterodimer content in such setup was chosen for the optimization of reagents and reaction conditions, as shown in Figure 1 and Figure 2 to ensure high performance also in suboptimal future binder combinations. DuoBody reactions were carried out in 2 mL Eppendorf vials by manual pipetting, at room temperature (RT), using a 1 mg/mL antibody concentration and volumes from 100 to 1000 µL for 0–18 h (Figure 1). TCEP (cat. no. 646547) and dhAA (cat. no. 261556), as well as PEG-azide (PEG-N_3_, cat. no. 901136), were from Sigma Aldrich, Saint Louis, MO, USA.

### 4.2. Miniaturized and Automated cFAE

For miniaturization, samples and reactants were combined to a total volume of 100 µL in 96-well microtiter plates (Eppendorf, Hamburg, Germany) with 1–0.1 mg/mL antibody concentrations (Figure 1 and Figure 2) and incubated at RT. To avoid extensive evaporation within the Hamilton robotic devices, 2 mL deep-well plates were evaluated after sealing with lids or X-pierce seals, but process realization on a Tecan device in a laboratory with low ventilation resulted in a neglectable volume reduction. Matrix pipetting was carried out applying a customized protocol on a Tecan device as well as a customized solution for bulk database upload and tracking of the combinations.

### 4.3. Analytical SE-HPLC

Size-exclusion HPLC (SE-HPLC) was run using a Bio Resolve SEC mAb column (Waters, Milford, MA, USA) and 50 mM sodium phosphate pH 6.3 with 0.4 M sodium perchlorate (Merck KGaA, Darmstadt, Germany) as the running buffer. We injected 7.5 µg of sample, and chromatography was run for 7 min at a flow rate of 0.35 mL/min, recording the absorbance at 214 nm wavelength. Supplementary Figure S1 contains detailed information on an early extra peak by PEG-azide that can be neglected for analyses.

### 4.4. Analytical HIC-HPLC

For hydrophobic interaction chromatography (HIC-HPLC), the samples were adjusted to an antibody concentration of 0.5 mg/mL and 0.5 M ammonium sulfate (Merck KgaA, Germany). Then, 80 µL (40 µg) was injected into a TSKgel Butyl NPR (Tosoh, Tokyo, Japan) column, and separation was performed using a full linear gradient from A (1.2 M ammonium sulfate (Merck KgaA, Germany), 1× PBS pH 7.2 (Invitrogen, Carlsbad, CA, USA)) to B (50% (*v*/*v*) methanol (VWR, Atlanta, GA, USA), 0.1× PBS, pH 7.2) within 15 min at a 0.5 mL/min flow rate, recording the absorption at 280 nm wavelength.

### 4.5. ADC Conjugation

ADCs were produced by enzymatic conjugation as described previously, utilizing microbial transglutaminase (MTG) and a valine–citrulline–MMAE (Moradec LLC, San Diego, CA, USA) linker–payload equipped with a primary amine [34]. The targeted drug-to-antibody ratio (DAR) was 4, based on the number of MTG conjugation sites: a transglutaminase recognition sequence (Qtag) attached to the C-terminus of each LC and the natural Q295 (EU numbering) position in the CH2-domain of the antibody. The antibodies were conjugated as described before [34] at a 2.5 mg scale for the test conjugations as well as at 20 mg for the preparation of the final batches and purified using preparative SEC with 1× PBS pH 7.0 as the running buffer. The samples were concentrated, sterile-filtered and analyzed via SE-HPLC, HIC-HPLC and RP-HPLC.

### 4.6. Half-Conjugated ADC DuoBody Reaction

The generation of biparatopic half ADCs was optimized by screening 24 different reaction conditions: 1 mg/mL or 2 mg/mL reaction concentration, 20- or 40-fold molar TCEP excess, and cFAE incubation times of 4 h, 6 h and 8 h. The samples were mixed in a 200 µL volume, followed by the addition of a 40-fold molar excess of PEG-azide (Sigma Aldrich, Saint Louis, MO, USA) over TCEP for quenching and incubation for 72 h at RT to allow sample reoxidation.

For the preparative reactions, 1 mg/mL of mAb/ADC was mixed with a 40-fold molar TCEP excess and incubated for 6 h, followed by the addition of PEG-azide in s 20-fold molar excess over TCEP and incubation for 120 h at RT.

### 4.7. Analytical CIEX-HPLC

DuoBody formation was evaluated via CIEX-HPLC using a MAbPac^TM^ SCX-10 HPLC column (Thermo Fisher Scientific, Waltham, MA, USA). For the reactions of unconjugated mAbs, a linear gradient starting from 97% buffer A (CX-1 pH Gradient Buffer A pH 5.6, Thermo Fisher Scientific, USA) and 3% buffer B (CX-1 pH Gradient Buffer B pH 10.2, Thermo Fisher Scientific, USA) up to 36% buffer A, 32% buffer B and 32% buffer C (1M sodium chloride, Merck KGaA, Germany) over 10 min was applied.

For ADC DuoBody reactions, chromatography was run at 1 mL/min with a linear gradient starting from 97% buffer A (10 mM sodium phosphate pH 5.6) and 3% buffer B (10 mM sodium phosphate pH 10.2) up to 50% buffer B and 50% buffer C (1 M sodium chloride) over 11 min. The flow rate was set to 1 mL/min, and absorption was recorded at 280 nm wavelength.

### 4.8. Cytotoxicity Evaluation

In vitro potency assessment of the ADCs was carried out on the c-MET-expressing human lung cancer cell lines NCI-H441 (ATCC) and EBC-1 (now National Institutes of Biomedical Innovation, Health and Nutrition, Japanese Cancer Research Bank). NCI-H441 cells were cultured in RPMI1640 medium with GlutaMAX TM supplement (Thermo Fisher Scientific, USA), 1 mM sodium pyruvate (Thermo Fisher Scientific, USA), 2.5 g/L of glucose (Sigma Aldrich, USA) and 10% FBS (Sigma Aldrich, USA), EBC-1 cells in MEM (Minimum Essential Eagle Medium, Sigma Aldrich, USA) with 2 mM glutamine (Thermo Fisher Scientific, USA) and 10% FBS (Sigma Aldrich, USA). The day before treatment, 1000 (A549), 2000 (EBC-1) or 3000 cells/well (NCI-H441) were seeded in 384-well flat-bottom microplates (Thermo Fisher Scientific, USA) and incubated in 5% CO_2_, at 37 °C. After sample addition and 6 days of incubation, 100 µL of CellTiter Glo^®^ (Promega, Madison, WI, USA) reagent was added, and the samples were mixed for 2 min under shaking at 300 rpm, followed by a 20 min incubation, protected from light, and the final measuring of luminescence using an EnVision 2104 multilabel reader (PerkinElmer, Waltham, MA, USA). The processed data were fit using the dose–response by %effect vs. concentration [M] with the equation log(inhibitor) vs. response–variable slope (four parameters) (GraphPad Prism version 8.2.0, GraphPad Software, Boston, MA, USA).

### 4.9. c-MET Degradation Screen

For the c-MET degradation screen, a Lumit quantification approach (Promega, cat. no. W1232) was optimized for the automated 384-well plate setup. In total, 5000 MKN-45 cells (DSMZ no.: ACC 409; cultured in 80% RPMI 1640, 2 mM Glutamax, 1 mM Na pyruvate, 20% FBS, 1% NEAA, all from Thermo Fisher Scientific, USA) with >95% viability were seeded in 20 µL in 384-well plates (Greiner, cat. no.781080, Kremsmuenster, Austria) using a Star liquid handler (Hamilton, Franklin, MA, USA) under a HEPA hood one day prior to the addition of 2 µL of sample using a Biomek I7 device and further incubation at 37 °C and 5% CO_2_ for additional 24 h. Assay controls were an in-house produced isotype control antibody, the in-house reproduced REGN5093 positive control and the luciferase inhibitor MSC2755228A-1. On day 3, 3 µL of lysis buffer (containing protease and phosphatase inhibitors, Millipore, cat. nos. 539134 and 524629, respectively) was added, followed by 30 min incubation at RT without shaking. Then, 12 µL of a detection antibody mix (anti-c-MET (D1C2) XP rabbit mAb and (L41G3) mouse mAb, Cell Signaling Technology, cat. nos. 8198S and 3148S, at 50 and 315 µg/mL, respectively) was added using a Multidrop Combi dispenser with subsequent incubation for 95 min at RT without shaking, before the addition of 6 µL of detection reagent (anti-mouse Ab Ab-SmBit and anti-rabbit Ab Ab-LgBit, Promega, cat. nos. W1052 and W1042) according to the manufacturer’s protocol. Luminescence was read out after 15 min on a Pherastar reader. The experiments were performed in triplicate. The data were used to describe the dose–response by %effect vs. concentration [M] with the equation log(inhibitor) vs. response–variable slope (four parameters) and to calculate IC50 values using GraphPad Prism software (Version 8.2.0).

## Data Availability

Data available upon reasonable request to the corresponding author.

## Supplementary Materials

The following supporting information can be downloaded at https://www.mdpi.com/article/10.3390/ijms25042097/s1.

## Abbreviations

2-MEA	2-mercaptoethylamine•HCl
ADC	antibody–drug conjugate
bpADCs	biparatopic antibody–drug conjugates
bsAb	bispecific antibody
cFAE	controlled Fab-arm exchange
CIEX	cation-exchange chromatography
dhAA	dehydroascorbic acid
Fc	fragment crystallizable
HIC	hydrophobic interaction chromatography
HTP	high throughput
HTS	high-throughput screening
IgG	immunoglobulin G
mAb	monoclonal antibody
MMAE	monomethyl auristatin E
MoA	mode of action
MW	mass weight
PEG-Azide	PEG-N_3_, polyethylene glycol–azide
PTM	post-translational modification
RP-HPLC	reverse-phase high-pressure liquid chromatography
TCEP	tris(2-carboxyethyl)phosphin
