# Supplementary material for: A Generic Approach for Miniaturized Unbiased High-Throughput Screens of Bispecific Antibodies and Biparatopic Antibody–Drug Conjugates
Source: Int J Mol Sci. 2024 Feb 8;25(4):2097. doi: 10.3390/ijms25042097 (PMC10889805; doi:10.3390/ijms25042097)
Supplement: Supplementary file 1 [file ijms-25-02097-s001.zip › ijms-2756908-supplementary.pdf]

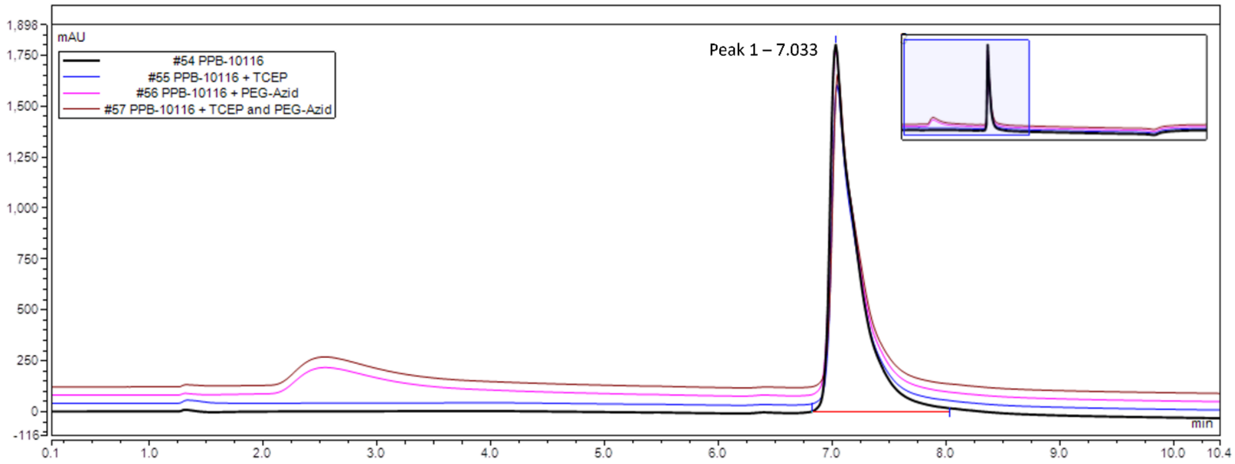

Figure S1. $PEG-N_3$ peak in analytical HIC chromatograms. A peak at 2 – 4 min is observed only in samples containing $PEG-N_3$ and therefore not integrated or used in calculations of heterodimer content throughout this study.

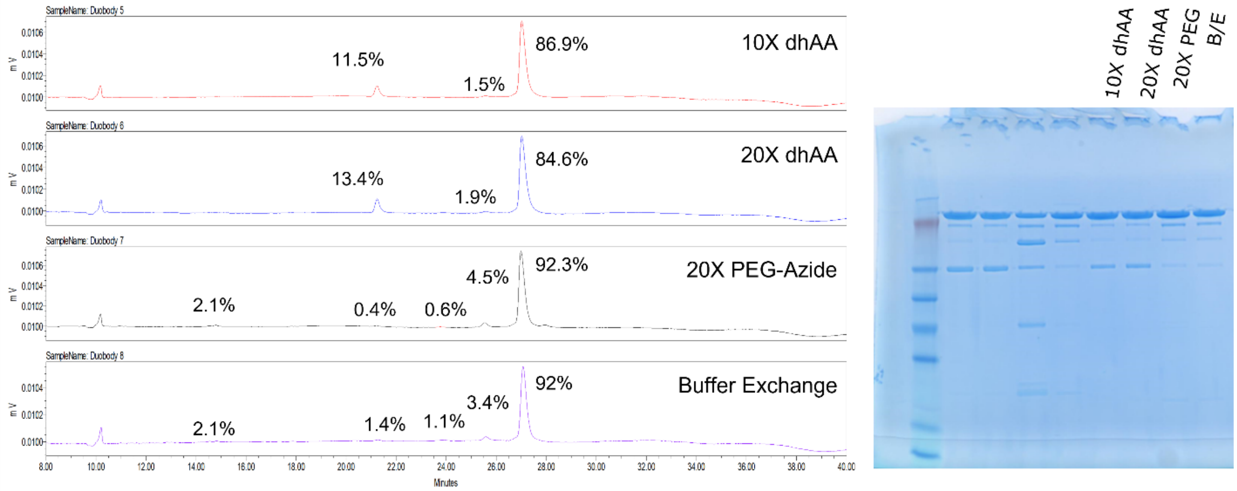

Figure S2. Analysis of re-oxidation. Left: CE-SDS quantification of of samples evaluated by SDS-PAGE shown in Figure 1d. Samples were evaluated after 3.5 days incubation at room temperature. Right: Uncropped SDS-PAGE gel as shown in Figure 1d.

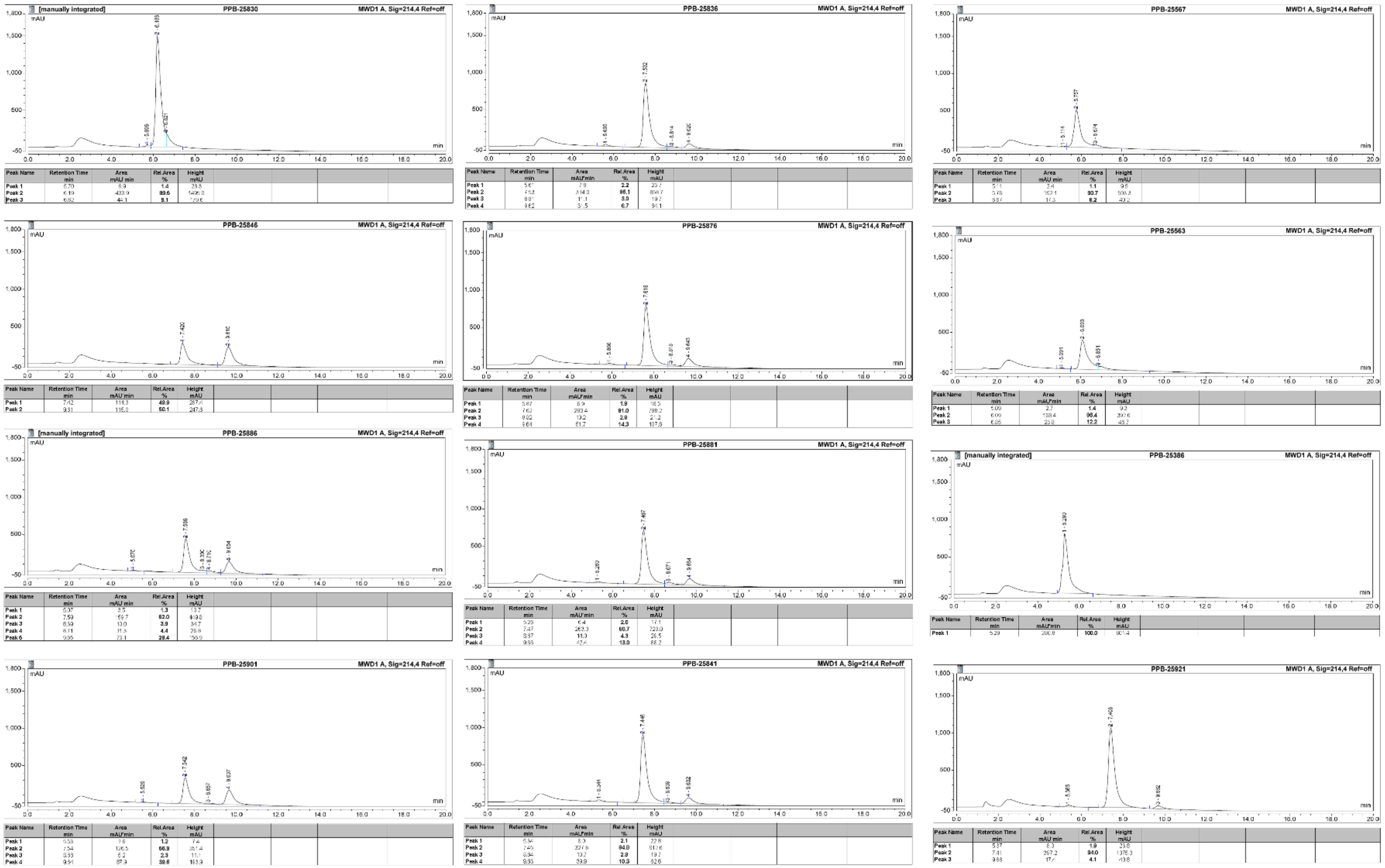

Figure S3. Randomly picked HIC chromatograms for 12 of 624 heterodimeric biparatopic antibodies. Upper left REGN5093 small scale re-production and heterodimerization. Further samples in left panel, as well as in lower percentage in middle panel, exhibit remaining excess of isotype control VHH-Fc. Large scale production vs small scale and different concentration determination may have led to imbalanced molar ratio of parentals. Not observed in samples combining anti-c-MET VHHs and Fabs (right panel).

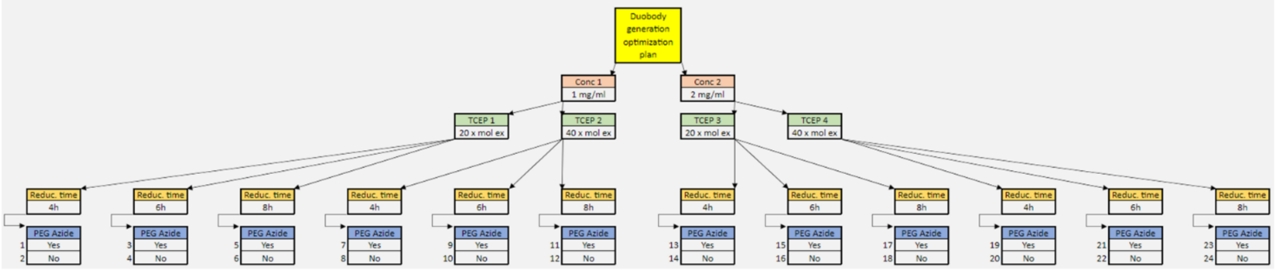

Sample	Plate position	Conc. [mg/ml]	TCEP eq.	Time [h]	PEG-Azide	Duobody [%]	ADC (F405L) [%]	IgG (K409R) [%]
49	E1	1	20	4	Yes	91.4	3.0	5.6
50	E2	1	20	6	Yes	92.3	3.2	4.5
51	E3	1	20	8	Yes	92.7	3.4	3.9
52	E4	1	40	4	Yes	92.9	3.5	3.7
53	E5	1	40	6	Yes	92.9	3.4	3.7
54	E6	1	40	8	Yes	93.6	3.4	3.0
55	E7	1	20	4	No	96.0	3.9	0.1
56	E8	1	20	6	No	95.8	4.2	0.1
57	E9	1	20	8	No	89.1	3.7	7.2
58	E10	1	40	4	No	95.9	3.9	0.2
59	E11	1	40	6	No	95.7	3.9	0.4
60	E12	1	40	8	No	94.1	3.8	2.1
61	F1	2	20	4	Yes	91.2	2.8	5.9
62	F2	2	20	6	Yes	92.6	3.1	4.3
63	F3	2	20	8	Yes	91.9	2.8	5.3
64	F4	2	40	4	Yes	91.3	2.9	5.8
65	F5	2	40	6	Yes	91.4	2.8	5.8
66	F6	2	40	8	Yes	91.9	2.3	5.8
67	F7	2	20	4	No	91.1	3.1	5.8
68	F8	2	20	6	No	92.0	3.5	4.5
69	F9	2	20	8	No	90.7	3.3	6.0
70	F10	2	40	4	No	95.5	3.1	1.4
71	F11	2	40	6	No	94.6	3.2	2.2
72	F12	2	40	8	No	95.8	3.1	1.1

Figure S4. Overview on heterodimer contents during process optimization for formation of half-ADCs

Table S1. Numerical data as shown in Figure 1b and Figure 1c.

Mol eq. TCEP	t [h]	Peak area [mAU*s]		
		Parental 1	Bispecific	Parental 2
2.5x	0	298.43	0.00	391.73
2.5x	1.5	231.93	57.21	350.98
2.5x	3	165.65	165.34	244.04
2.5x	4.25	127.71	247.13	232.31
2.5x	5.75	103.50	295.70	155.72
2.5x	7	90.27	333.83	136.30
5x	0	275.64	0.00	382.48
5x	1.5	177.91	130.89	284.45
5x	3	82.23	294.74	168.38
5x	4.25	46.19	387.62	173.35
5x	5.75	29.30	422.23	163.39
5x	7	23.03	440.52	173.65
10x	0	265.58	0.00	367.26
10x	1.5	105.67	233.65	185.03
10x	3	32.12	387.28	120.73
10x	4.25	18.17	420.60	99.23

10x	5.75	13.42	434.16	94.68
10x	7	12.17	440.68	104.50
20x	0	269.28	0.00	371.33
20x	1.5	91.88	293.22	175.01
20x	3	19.57	437.11	112.32
20x	4.25	15.30	478.89	117.78
20x	5.75	17.93	473.72	96.86
20x	7	7.17	469.66	97.49

Table S2. Numerical data as shown in Figure 2b.

		Peak area [mAU*s]		
c (mAb)	t [h]	Parental 1	Bispecific	Parental 2
0.1 mg/ml	0	896.90	0.00	756.06
0.1 mg/ml	2	670.98	455.62	458.93
0.1 mg/ml	4	348.89	1018.85	197.61
0.1 mg/ml	6	195.13	1268.09	86.55
0.1 mg/ml	8	137.07	1348.71	36.21
0.1 mg/ml	10	36.21	1377.77	21.61
0.1 mg/ml	12	106.24	1382.37	11.52
0.1 mg/ml	14	103.53	1380.93	9.91
0.3 mg/ml	0	2749.05	0.00	2236.66
0.3 mg/ml	2	1010.12	3481.14	590.17
0.3 mg/ml	4	350.24	4508.16	133.87
0.3 mg/ml	6	268.04	4656.50	98.43
0.3 mg/ml	8	254.09	4429.56	10.96
0.3 mg/ml	10	250.15	4354.29	6.40
0.3 mg/ml	12	248.58	4356.09	9.06
0.3 mg/ml	14	247.06	4340.44	6.82
